# Improving outcomes of preschool language delay in the community: protocol for the *Language for Learning* randomised controlled trial

**DOI:** 10.1186/1471-2431-12-96

**Published:** 2012-07-09

**Authors:** Melissa Wake, Penny Levickis, Sherryn Tobin, Naomi Zens, James Law, Lisa Gold, Obioha C Ukoumunne, Sharon Goldfeld, Ha ND Le, Jemma Skeat, Sheena Reilly

**Affiliations:** 1 Centre for Community Child Health, Royal Children’s Hospital, Parkville, Australia; 2 Murdoch Childrens Research Institute, Parkville, Australia; 3 Department of Paediatrics, The University of Melbourne, Parkville, Australia; 4 Institute of Health and Society, School of Education, Communication and Language Sciences, University of Newcastle, Newcastle, United Kingdom; 5 Deakin Health Economics, Deakin University, Melbourne, Australia; 6 PenCLAHRC Peninsula College of Medicine and Dentistry, University of Exeter, Exeter, United Kingdom

**Keywords:** Language development, Mass screening, Language development disorders, Early intervention, Outcome assessment, Child development, Randomized controlled trial, Population characteristics

## Abstract

****Background**:**

Early language delay is a high-prevalence condition of concern to parents and professionals. It may result in lifelong deficits not only in language function, but also in social, emotional/behavioural, academic and economic well-being. Such delays can lead to considerable costs to the individual, the family and to society more widely. The *Language for Learning* trial tests a population-based intervention in 4 year olds with measured language delay, to determine (1) if it improves language and associated outcomes at ages 5 and 6 years and (2) its cost-effectiveness for families and the health care system.

****Methods/Design**:**

A large-scale randomised trial of a year-long intervention targeting preschoolers with language delay, nested within a well-documented, prospective, population-based cohort of 1464 children in Melbourne, Australia. All children received a 1.25-1.5 hour formal language assessment at their 4^th^ birthday. The 200 children with expressive and/or receptive language scores more than 1.25 standard deviations below the mean were randomised into intervention or ‘usual care’ control arms. The 20-session intervention program comprises 18 one-hour home-based therapeutic sessions in three 6-week blocks, an outcome assessment, and a final feed-back/forward planning session. The therapy utilises a ‘step up-step down’ therapeutic approach depending on the child’s language profile, severity and progress, with standardised, manualised activities covering the four language development domains of: vocabulary and grammar; narrative skills; comprehension monitoring; and phonological awareness/pre-literacy skills. Blinded follow-up assessments at ages 5 and 6 years measure the primary outcome of receptive and expressive language, and secondary outcomes of vocabulary, narrative, and phonological skills.

****Discussion**:**

A key strength of this robust study is the implementation of a therapeutic framework that provides a standardised yet tailored approach for each child, with a focus on specific language domains known to be associated with later language and literacy. The trial responds to identified evidence gaps, has outcomes of direct relevance to families and the community, includes a well-developed economic analysis, and has the potential to improve long-term consequences of early language delay within a public health framework.

****Trial registration**:**

Current Controlled Trials ISRCTN03981121.

## **Background**

### **Importance of language delay**

Children who have delayed language development as they move into school are at risk of a raft of difficulties. Impacts of poor oral language skills go well beyond early literacy development and ‘school readiness’ to increasingly apparent associations with emotional, behavioural and social difficulties [1-4]. Perhaps most worrying is the emerging evidence of very long-term sequelae that are not restricted solely to the school years or to children with serious clinical presentations [5]. Thus, epidemiologic data from the 1970 British Cohort Study show that difficulties at school entry have effects into adulthood on literacy, mental health and unemployment [6].

### **Epidemiology**

Language delay is one of the most common pre-school developmental difficulties. Prevalence estimates vary according to definition and cut point. The most quoted estimate (7% of 5 year olds) includes only those with *specific language impairment*, a specific category of children demonstrating poor language skills but with normal non-verbal cognition [7]. The prevalence is higher when criteria include all children with language delay; for instance, in our *Early Language in Victoria Study* (ELVS), nearly 20% of 4 year olds scored below −1.25 standard deviations (SD) and 25% below −1 SD on one or both of the standardised expressive and receptive (comprehension) axes [8]. Rates are even higher in socially disadvantaged populations, with language delay affecting up to 50% of preschool children reared in poverty [9]. While SLI criteria are often used in clinical research, there is evidence that language and cognition share their genetic foundation, [10] and that children with and without specific delay have broadly the same overt language features and need for intervention services [11].

### **Does intervention improve language outcomes?**

There are growing grounds for optimism that interventions can improve language delay. Between publication in 2003 and the 2012 update, the number of trials included in the Cochrane review of interventions for children with speech and language delays/disorders rose from 33 to 64, with the number of trials included in meta-analysis rising from 25 to 54 with a total of 3872 participants [12]. Positive outcomes were identified in a number of areas, notably expressive vocabulary (effect size 0.7, 95% confidence interval (CI) 0.05 to 1.25, p = 0.04), syntax (effect size 0.6, 95% CI 0.15 to 1.95, p = 0.01) and overall phonological development (effect size 0.4, 95% CI 0.13 to 0.72, p = 0.005). Less attention has been paid to broader social outcomes, such as the impact of interventions on activity limitation and participation restriction, that are of critical importance both for children’s development and success [13] and for families [14].

The majority of these trials could be construed as “targeted” interventions delivered by speech and language therapists. Parent training is a feature of a number of the studies of younger children but it is rarely possible to distil the discrete effect of parental input. Some of the review’s studies include intervention delivered by less specialised staff, early educationalists, paraprofessionals etc. under the guidance of a speech and language therapist, but to date a clear picture has not emerged as to whether such interventions, while presumably cheaper, are as effective as those delivered by specialists.

While encouraging, this evidence remains limited. Most studies are small (under 20 in each arm) with limited follow-up, many of the studies are not protocol driven and detail of the interventions was often lacking. Unsurprisingly there are few replications and heterogeneity is high. Most of the studies were ‘efficacy’ trials, carried out in controlled environments with therapies often administered by the person developing the intervention. Very few could be construed as ‘effectiveness’ trials with the potential to be rolled out across a service. The underlying populations were often not well-characterised, and little is ever reported about the children’s developmental history. Finally, very few of these studies included any form of economic analysis, making it impossible to establish the costs and benefits of the interventions.

### **Taking language intervention to the population level**

Given both the prevalence and long-term consequences of early language delay, it is clearly an important public health issue and one for which the development of appropriate, effective population-level interventions has the potential to make a major contribution to society [15,16].

However, the optimal timing to intervene for language delay in whole populations is not known. Very early intervention for children with late-onset language may be inefficient, because most such children resolve spontaneously by ages 3 [17] and 4 years [18]. It is now apparent that false negatives are very common up to at least 3 years. Thus, in the Early Language in Victoria Study, around half of those children formally assessed as having language delay at age 4 were not late talkers at age 2 [8].

Conversely, there are also questions about the efficacy of interventions after school commencement. The only rigorous, large-scale trial to date involved 161 6–11 year olds with language delay; short-term benefits for intervention children receiving intensive speech/language therapy over 6 months were not sustained to 12 months [19]. While this might relate to the nature of this specific intervention, alternatively it might mean that language delay is already relatively ‘fixed’ in older children.

### **Designing an effective population approach - the next steps**

Given the evidence above that treatment *can* be effective, the next step is to determine whether systematic delivery of effective treatment *does* deliver substantial population benefit. If 2–3 years is too early but the school years too late, 4–5 years may represent an ideal window. Yet by 4 years language delay is clearly not homogeneous, so therapy must be flexible enough to meet individual needs [20].

The trial reported here is designed to translate the available evidence into a program that addresses many of the issues discussed above. Such a program would ideally be standardised and replicable, yet flexible enough for children with diverse cognitive and language profiles (unlike the standardised, uniform programs currently the focus of population research at younger ages, eg [21,22]). It would be of greater intensity and duration than achieved in most clinical services, [23] since the limited literature suggests a dose–response relationship [24] with both duration [25] and intensity [26]. This must, however, be weighed against cost and logistic constraints, as well as parent priorities. In our trials, parents have been willing to attend blocks of up to 6 sessions for child issues detected by screening that they consider important but not necessarily urgent, such as overweight [27,28] and slowness to talk [22].

Boyle’s large-scale RCT in 6–11 year old children, although ultimately ineffective, did demonstrate that a flexible, intensive (1.5-2 hours per week over 15 weeks), replicable, manual-guided therapy program is feasible for use with large numbers of language-delayed children [19]. The intervention was designed along dimensions previously identified for manual-guided treatment, [29] drew together procedures for language intervention considered by researcher and professionals likely to be effective, [30] and was well-received by children, schools and parents.

In light of the above, we are therefore conducting a novel population-based trial of intervention for language delay at age 4. Designed to address the identified evidence gaps, it will have adequate statistical power on the available evidence. It is manual-driven to be standardised yet flexible, is designed so that it could be rolled out in the community, will have outcomes of direct relevance to the families and the community, and includes a well-developed economic analysis. Because the participating children have been followed since infancy (see below), a rich early dataset is available with which to explore differential impacts of the intervention.

### **Aims and hypotheses**

The *Language for Learning* trial poses two specific researchable questions:

1. Does a population-based intervention targeting 4 year olds with language delay (expressive and/or receptive standard scores more than 1.25 standard deviations below the mean) improve language and associated outcomes?

2. Is the intervention cost-effective for families and the health care system?

We **hypothesise** that:

1. Compared to the control group, benefits to the intervention group at 5 and 6 years will include better mean scores on standardised tests of:

a. Expressive/receptive language (primary functional outcomes) and vocabulary, phonological analysis and narrative skills

b. Other secondary outcomes:

i. Social skills and relationships

ii. Emotional and behavioural well-being

iii. Early literacy

iv. Health-related quality of life

v. ‘School readiness’, measured by the Australian Early Development Index (AEDI)

2) The intervention will be acceptable and cost-effective (against common decision thresholds).

## **Methods/design**

### **Study design**

**Figure 1:**
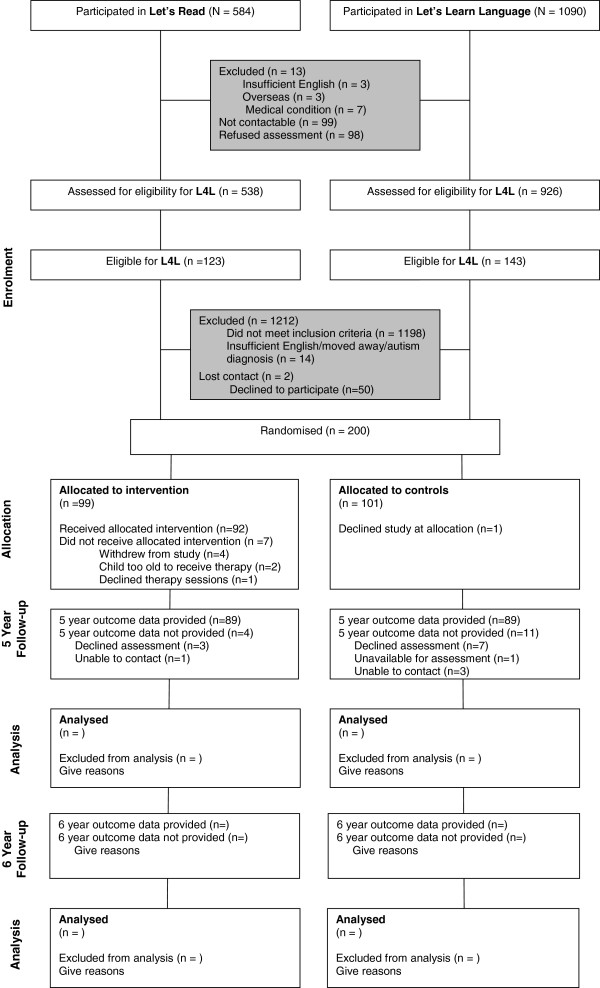
**Participant flow-chart for *Language for Learning***.

*Language for Learning* is a large-scale randomised trial (ISRCTN03981121) of a targeted year-long intervention for expressive and/or receptive language delay at age 4 years, nested within a cross-sectional population-based ascertainment of language delay and described here in accordance with CONSORT guidelines. Figure 1 shows progress at time of writing. Because it has re-recruited participants from two earlier low-intensity language and literacy promotion trials with null findings, *Let’s Learn Language*[17] and *Let’s Read*[31], the trial is taking place predominantly in the same 8 Melbourne local government areas (LGAs) in which these participants continue to reside.

### **Prior research with this sample**

Children in these two completed trials turned 4 in 2010. Combining these samples provided efficiencies in time and cost, as well as providing a wealth of early-life data on the participants and their prior service utilisation (see Measures, below). Features common to both trials include: (1) their population focus, targeting all children born in defined periods in 8 of Melbourne’s 31 LGAs (each mean birth rate, 1400 per annum); (2) recruitment in infancy by Maternal & Child Health (M&CH) nurses, who provide well-child care to all Victorian children to age 5 (reach: 97% after birth and≈75% at 12 months); (3) repeated annual measures of children’s language, behaviour and potential confounders, with many measures common to both trials; and (4) very high retention rates.

Briefly, the *Let’s Read* trial (ISRCTN04602902) aimed to determine whether a shared book-reading intervention delivered universally through primary care over the first 3 years of life improved language and pre-literacy outcomes by age 4 years. In 2006, M&CH centres in 5 relatively disadvantaged LGAs recruited around 650 infants at age 4–8 weeks. After subsequent randomisation intervention nurses then delivered four brief (10 minute) literacy promotion interventions at the routine 4–8, 12 and 18 month and 3½ year old visits that are part of the well-child care schedule available to every child born in Victoria. Despite excellent uptake and 89% retention at 4 years, intervention and control children had similar language and preliteracy outcomes at age 4 years [32]. Further, although recruited from relatively disadvantaged areas, the participants themselves were not particularly disadvantaged.

The *Let’s Learn Language* trial (ISRCTN20953675) aimed to determine whether a 6-week group parent language promotion program for slow-to-talk toddlers improves language at 2 and 3 years. 1217 children were recruited at 12 month M&CH visits in 2007; the 301 scoring ≤ 20th percentile on a 100-word expressive vocabulary list at 18 months entered the trial. Again, despite extremely good uptake and 89% retention at 3 years, intervention and control children had similar expressive and receptive language, vocabulary and behaviour at age 3, and language scores were very close to those of the general population [17].

### **Summary of procedures**

**Figure 2:**
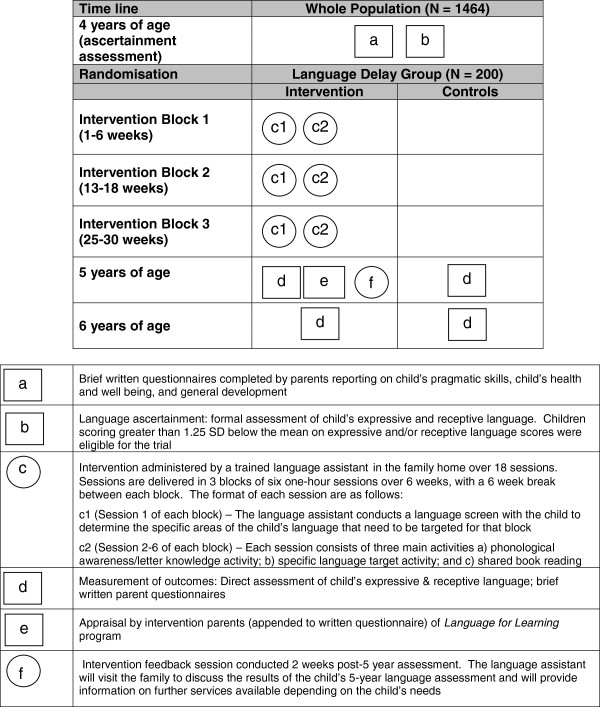
**Pictorial diagram of *Language for Learning *trial**.

Figure 2 graphically summarises the trial and its procedures for both the intervention and control groups in the form of a Perera diagram [33]. Two weeks before each child’s 4^th^ birthday, parents of each *Let’s Read* and *Let’s Learn Language* participant were re-contacted and invited to participate in the new trial. Parents were sent brief written questionnaires and children received formal language assessments (*Let’s Read* children, March-July; *Let’s Learn Language* children, May-December 2010). Eligible children who entered the trial were then randomised (see below), with intervention children then offered a 20-session intervention program that ran between the 4^th^ and 5^th^ birthdays. All children are being re-assessed at 5 and 6 years by researchers blind to randomisation status at a single face-to-face visit in the child’s home or a convenient local venue (e.g., their maternal and child health centre).

### **Inclusion criteria**

Children were eligible for the trial if they participated in either the *Let’s Read* or *Let’s Learn Language* trials, and had expressive and/or receptive language scores more than 1.25 SD below the normative mean on the CELF-P2 at age 4 years, with no child younger than 4.0 years, and no child older than 4.8 years at assessment.

### **Exclusion criteria**

Were known intellectual disability, major medical conditions, hearing loss >40 dB HL in the better ear and autism spectrum disorders. Children for whom English is a second language were not excluded, but parents had to be able to complete questionnaires without interpreters at a Grade 6 level of written English.

### **Randomisation**

OU (biostatistician) coordinated the randomisation process. Allocation to the trial arms, via computer generated random numbers, was concealed using sealed opaque envelopes. Envelopes were ordered and opened only upon confirmation of consent and recruitment for each participant. Randomisation was stratified by prior trial participation (*Let’s Read* versus *Let’s Learn Language*) and modality of language problem at recruitment (receptive only, expressive only, or both receptive and expressive). Randomisation was blocked within each stratum using randomly-permuted block sizes in a non-systematic sequence. The randomisation sequence was held by a researcher otherwise unconnected to the trial who revealed each child’s allocation to trial staff upon confirmation of recruitment.

Parents were mailed letters telling them of their child’s allocation status. Control group letters outlined the child’s language status and available speech pathology services, using letters developed for the *Early Language in Victoria Study* in 2007 and approved by the RCH Ethics Committee. Intervention group letters included information about the intervention program. Trial staff then phoned the parent to answer questions and arrange the first sessions.

### **Intervention overview**

Each child commenced the 20-session, year-long program within approximately 2 months of the 4-year-old baseline. It comprises 18 weekly intervention-focused sessions in three 6-week blocks starting every 3 months; the 5-year-old blinded assessment; and an exit feedback/planning session in the following month (see Figure 2). Sessions are delivered in the child’s home by a ‘language assistant’, a university graduate experienced with parents and children and knowledgeable about child health and development; while we did not specify the professional background, the assistants ultimately had psychology and sociology backgrounds.

Before the intervention commenced, the language assistants were trained in the program, its activities and manual, and in maximising the ‘therapeutic alliance’. The pre-intervention training consisted of a ½-day workshop as well as a 2-hour long 1:1 session with the speech pathologist. Two further ½-day workshops were held before the commencement of the second intervention block (focusing on intervention techniques) and after Block 3 (focusing on the 5 year follow up assessments and the procedures and content for session 20).

Although the content is specific to this project, the manual design was adapted from Boyle, [19] and explains program principles and domains, documents the standardised activities and their hierarchies, and provides brief tasks to monitor the child’s progress towards their individual goals. The session structure was based on the ‘emergent literacy intervention program’, [34] where each session follows a specific pattern and includes a focus on alphabet knowledge and narrative.

At each session, the child and parent receive an hour of one-on-one contact with the language assistant, including: (1) brief review; (2) activities introduced by the language assistant directed at the child; (3) activities for parent and child together, with support from the language assistant; and (4) activities for home practice. Although each activity has standardised supporting materials and manual instructions, the program is personalized by selecting harder or easier (‘step up’ and ‘step down’) activities according to profile, severity and progress. In addition to the standard components of narrative skills and phonological awareness/pre-literacy skills (see below: Intervention content), specific activities are selected for each child for each block from the range offered in the manual (see below: Intervention content -Vocabulary and grammar) depending on the child’s language skills. Before each 6-week block, the trial’s coordinating speech pathologist (NZ) and each language assistant review progress and jointly select the mix and level of activities.

Four basic principles are followed. Therapy directly and overtly targets parent participation; it provides varied activities and offers multiple opportunities for practising each skill; activities are child- and parent-friendly and fun; and it encourages good parent–child interaction strategies, e.g., reinforcement and praise, following the child’s conversational lead, and ‘scaffolding’ child attempts.

### **Intervention content**

Sessions include activities that encompass three domains, chosen for their importance to language, social and educational outcomes and demonstrated feasibility for standardised large-scale intervention delivered without specialised speech pathologist skills [19].

*Vocabulary and grammar* deficits impact on language, literacy [35] and discourse/narrative skills. Both are crucial for social and educational attainment and considered ameliorable [24]. Depending on the child’s language skills, activities focus on vocabulary expansion (e.g., learning new words), identifying word features (e.g., semantic groups such as ‘animals’), sentence structures and grammatical markers (e.g., targeting correct sentences or ‘ing’ endings in verbs), or comprehension skills (e.g., following instructions and asking clarifying questions if needed). With support from the language assistant, these vocabulary and grammar activities are directed at the parent and child together.

*Narrative skills,* often a focus for clinical intervention, [36] underpin communicative competence and correlate strongly with reading comprehension; deficits impact on social interaction and understanding of classroom processes [37]. In this intervention program, they are targeted through shared book reading activities, which explicitly teach ‘story grammar’ elements such as ‘who, what, where’ [37].

*Phonological awareness/preliteracy skills* are strongly linked to oral language [38] and literacy, [39] are usually established well before a child starts school, [40] and can be effectively taught to children with language disorders [41]. Print conventions (e.g., left to right reading), awareness of rhyme, and letter-sound connections are targeted through shared book reading. Other activities specifically target skills like phoneme identity and phoneme matching and are directly taught to the children by the language assistant.

### **Measures**

*4 year old ascertainment assessment (4 years, n≈1,500):* Because of uncertainties noted in all systematic reviews about the predictive properties of screening tools, [24,42,43] the main criterion for study entry was a formal assessment of language skills using the Clinical Evaluation of Language Fundamentals-Preschool (CELF-P2) [44]. The CELF-P2 is norm-referenced for children from 3:0–6:11 years and yields two core subscales of receptive and expressive language. We administered the Word Structure, Expressive Vocabulary and Recalling Sentences Expressive subtests and the Sentence Structure, Concepts and Following Directions and Basic Concepts Receptive subtests. We did not analyse the Language Content or Language Structure Indices.

Trial measures collected at 4 years (baseline), 5 years (intermediate outcomes) and 6 years (definitive outcomes) are detailed in Table 1. A range of measures (not described here) were also collected at multiple waves between 1 and 3 years of age in the two preceding trials, and will support additional exploratory and mediator analyses.

**Table 1:** **Primary and secondary outcome measures for the *Language for Learning *Trial**

**Construct**	**Timing (years)**			**Measure**	**Additional information**
	**4**	**5**	**6**		
**Primary Outcome**					
Expressive and Receptive language	▪	▪	▪	Clinical Ev aluation of Language Fundamentals - Preschool Second Edition (CELF P2) [44]	Baseline only: Basic Concepts
					
					5 Years only: Word Classes (receptive + expressive subtests)
					6 Years only: Word Classes (receptive only).
**Secondary Outcomes**					
Receptive vocabulary	▪		▪	Peabody Picture Vocabulary Test (PPVT-4) [45]	Used with Intervention group only at 4–5 Years (Baseline)
Phonological skills		▪	▪	Comprehensive Test of Phonological Processing (CTOPP) [46]	Subtests used: Elision; Blending Words; Sound Matching.
					Contribute to Phonological Awareness Composite Score.
	▪	▪		Sutherland Phonological Awareness Test – Revised: Modified (SPAT-R) [47]	Three individual scores obtained for the 3 subtests used: Rhyme Detection Subtest; Onset Phoneme Identification; Letter Knowledge (study specific)
	▪		▪	Children's Test of Non-Word Repetition (CNREP) [48]	Data on subgroup only at baseline, as measure was discontinued due to time restrictions
Literacy skills			▪	Wide Range Achievement Test (WRAT) [49]	3 subtests used: Word Reading; Sentence Comprehension;
					Spelling; Word Reading and Spelling create ‘Reading Composite’
Pragmatic skills (social language use)	▪	▪	▪	Children’s Communication Checklist, 2^nd^ Edition (CCC:2) [50]	28 items on the 4 subscales used: inappropriate initiation; stereotyped language; use of context; nonverbal communication.
Narrative	▪		▪	The Renfrew Language Scales: Bus Story Test [51]	Used with Intervention group only at baseline
Non-verbal intelligence	▪			Kaufman Brief Intelligence Test, 2^nd^ Edition (KBIT-2) [52]	Only the matrices subtest was used as it gives a measure of nonverbal (fluid) intelligence
Early childhood development	▪	▪		Ages & Stages Questionnaire (A&SQ) [53]	5 developmental areas: Communication; Gross Motor; Fine Motor; Problem Solving; Personal-Social; totals for each developmental area are compared with empirical cut-points for each area
			▪	Australian Early Development Index (AEDI) [54]	Teacher reported questionnaire measuring 5 domains: social competence; emotional maturity; language and cognitive skills (school-based); communication skills and general knowledge
Quality of life	▪	▪	▪	Health Utilities Index (HUI)- Mark 2 and 3 [55]	Parent-reported measure scored using a single- and multi-attribute utility function based on preference scores (sensation, mobility, self-care, fertility, vision, hearing, speech, ambulation, dexterity, emotion, cognition, pain). Scores will be used to calculate quality-adjusted life years (QALYs)
	▪	▪	▪	Pediatric Quality of Life Inventory (PedsQL); parent-proxy [56]	Parent-completed 23 item scale comprising 4 dimensions, with 3 summary Scores: Total; Physical Health; Psychosocial Health.
Behaviour	▪	▪	▪	Strengths and Difficulties Questionnaire (SDQ ) [57]	25 item measure that yields one score of total behavioural problems and scores for emotional symptoms, conduct problems, hyperactivity, peer problems, and prosocial subscales
Service Utilisation	▪	▪	▪	Study generated	Parent-reported questionnaire to track health service utilisation by population with a specific health condition

### **Economic evaluation**

Although progress has been made in modelling the costs and long terms benefits of intervention for language-impaired children [58] and in interpreting unit costs, [59] economic analyses remain few and far between [60]. We will employ cost-consequences analysis conducted from both the broad societal perspective and the narrower perspective of the health care sector, [61] as interventions cost-effective from a health care perspective can add substantially to family costs [62]. The economic evaluation will compare any incremental costs of the intervention (costs accrued in the intervention arm compared to costs accrued in the control arm) to the full list of incremental primary and secondary outcomes, all expressed in their natural units of measurement. Uncertainty in the cost and outcome data and sensitivity of economic evaluation results to the methods of evaluation chosen will be tested through extensive sensitivity analyses.

The estimation of costs will collect resource use data from three main sources: research team records; intervention provider records; and parental report (via written questionnaires at child ages 4, 5 and 6). Key costs for the economic evaluation are program costs (including language assistant and other researcher time in relation to the intervention, intervention material costs and travel expenses) and family costs (family time spent on the intervention, costs to health service use and other government services outside of the intervention, and travel costs). Parents will be asked to recall health service resource use over the previous 12 months for their child, including doctor visits, other government services, private speech pathology, parental time and travel costs. Measured resource use will be valued using existing estimates of the cost of each unit of resource use from sources such as the Medicare Benefit Schedule fee rates for family practitioner and specialist doctor attendances, Australian Bureau of Statistics estimates of average Australian earnings, Royal Automobile Club of Victoria (RACV) estimates of travel costs, etc.

### **Sample size**

We anticipated that 1500 of the 1850 children in the two trials would be assessed at 4 years, allowing for 20% loss to follow-up (similar to ELVS). Assuming 240 subjects (16% of the 1500) would have language scores more than 1.25 SD below the CELF-P2 normative means, a further 10% would decline participation and 1% would be excluded, we estimated 210 subjects would enter the trial (105 in each arm). 10% attrition (similar to our recent behaviour [63] and obesity [28,64] trials) would provide 94 children in each trial arm at outcome, giving 80% power to detect a difference of 0.41 SD at the 5% level of significance. Even if attrition were 25%, we could still detect a 0.45 SD difference with 78 in each arm.

### **Data analysis**

For Hypotheses 1 and 2, outcomes and costs will be compared between the trial arms using the intention-to-treat principle with participants analysed according to the trial arms they were randomised to. We will compare mean outcomes at 5 and 6 year old follow-up using linear regression in unadjusted analyses and analyses adjusted for the following prognostic factors: child gender, whether recruited from *Let’s Read* or *Let’s Learn Language*, expressive and receptive language scores at baseline, and baseline measure of the outcome being considered when available.

The trial is powered primarily to address the main comparison between trial arms, but we will also use tests of interaction to conduct exploratory analyses addressing differential effects of the intervention across the following subgroups:

1. Language delay sub-group (expressive, receptive, mixed expressive/receptive);

2. Non-specific (non-verbal IQ < 85) vs specific (non-verbal IQ ≥ 85) language delay;

3. Social disadvantage, to determine whether this population intervention may increase, not decrease, inequalities.

Recognising that definitive answers to these complex issues may need even larger samples, we plan to make these data available for data pooling and meta-analysis via the Centre for Research Excellence in Children’s Language (NHMRC Grant 1023493) for which Reilly, Wake, Law, Gold, and Goldfeld are Chief Investigators.

## **Discussion**

This rigorous trial addresses the urgent need to improve the long-term consequences of early language delay, within a public health framework appropriate to its high prevalence and societal burden. Using existing cohorts offers time- and cost-efficiencies and a unique opportunity to understand different responses to therapy. The therapy interventions are not controversial, being already widely used clinically by speech pathologists. The flexible but standardised approach has already been shown by Boyle to be feasible and acceptable to parents and older children. Our innovation is in the systematic identification of language delay and rigorous attention to program delivery and dose in preschool children. The trial responds to identified evidence gaps, has outcomes of direct relevance to families and the community, and includes a well-developed economic analysis.

If effective, we expect the following outcomes:

•The best evidence yet that language delay can be readily identified, cost-efficiently addressed and significantly improved before formal schooling starts.

•A well-tested intervention that could potentially be delivered to children by a range of health and educational professionals, going some way to addressing the speech therapist shortages in a number of English-speaking countries and addressing a real and timely health services policy imperative.

## **Abbreviations**

AEDI: Australian early development index; A&SQ: Ages & stages questionnaire; CCC:2: Children communication checklist, 2^nd^ edition; CNREP: Children's test of non-word repetition; CELF-P2: Clinical evaluation of language fundamentals- preschool edition 2; CI: Confidence interval; CTOPP: Comprehensive test of phonological processing; dB HL: Decibels hearing loss; ELVS: Early language in victoria study; GSV: Growth scale value; HUI: Health utilities index; IQ: Intelligence quotient; KBIT-2: Kaufman brief intelligence test second edition; LGAs: Local government areas; M&CH: Maternal & child health; NHMRC: National health and medical research council; NCEs: Normal curve equivalents; PPVT-4: Peabody picture vocabulary test; PedsQL: Pediatric quality of life inventory; QALYs: Quality-adjusted life years; RCT: Randomised controlled trial; RACV: Royal automobile club of victoria; SLI: Specific language impairment; SD: Standard deviations; SDQ: Strengths and difficulties questionnaire; SPAT-R: Sutherland phonological awareness test – revised: modified; WRAT: Wide range achievement test.

## **Competing interests**

All authors declare that they and their spouses, partners or children have no financial and non-financial relationships or interests that may be relevant to the submitted work. The authors declare they have no competing interests.

## **Authors’ contributions**

MW conceived the *Language for Learning* trial with JL, LG, UO, SG, SR and JS; she takes overall responsibility for all aspects of the trial and this manuscript. ST was the Project Manager, assisted by PL and NZ. NZ and JL designed the intervention, with advice from JS and SR, who also advised on measures and their interpretation. OU advised on statistical issues, LG and HL on the economic evaluation, and SG on the translational aspects of the trial. All authors contributed, read and approved the final manuscript.

## Pre-publication history

The pre-publication history for this paper can be accessed here:

http://www.biomedcentral.com/1471-2431/12/96/prepub
